# Supplementary material for: The interactome of intact mitochondria by cross-linking mass spectrometry provides evidence for coexisting respiratory supercomplexes
Source: Mol Cell Proteomics. 2017 Dec 8;17(2):216–32. doi: 10.1074/mcp.RA117.000470 (PMC5795388; doi:10.1074/mcp.RA117.000470)
Supplement: Supplemental Data [file supp_RA117.000470_133922_0_supp_23978_fzffwf.zip › spectra_annotation/mito_spectra_annotation/181-1-2-1-20-1.pdf]

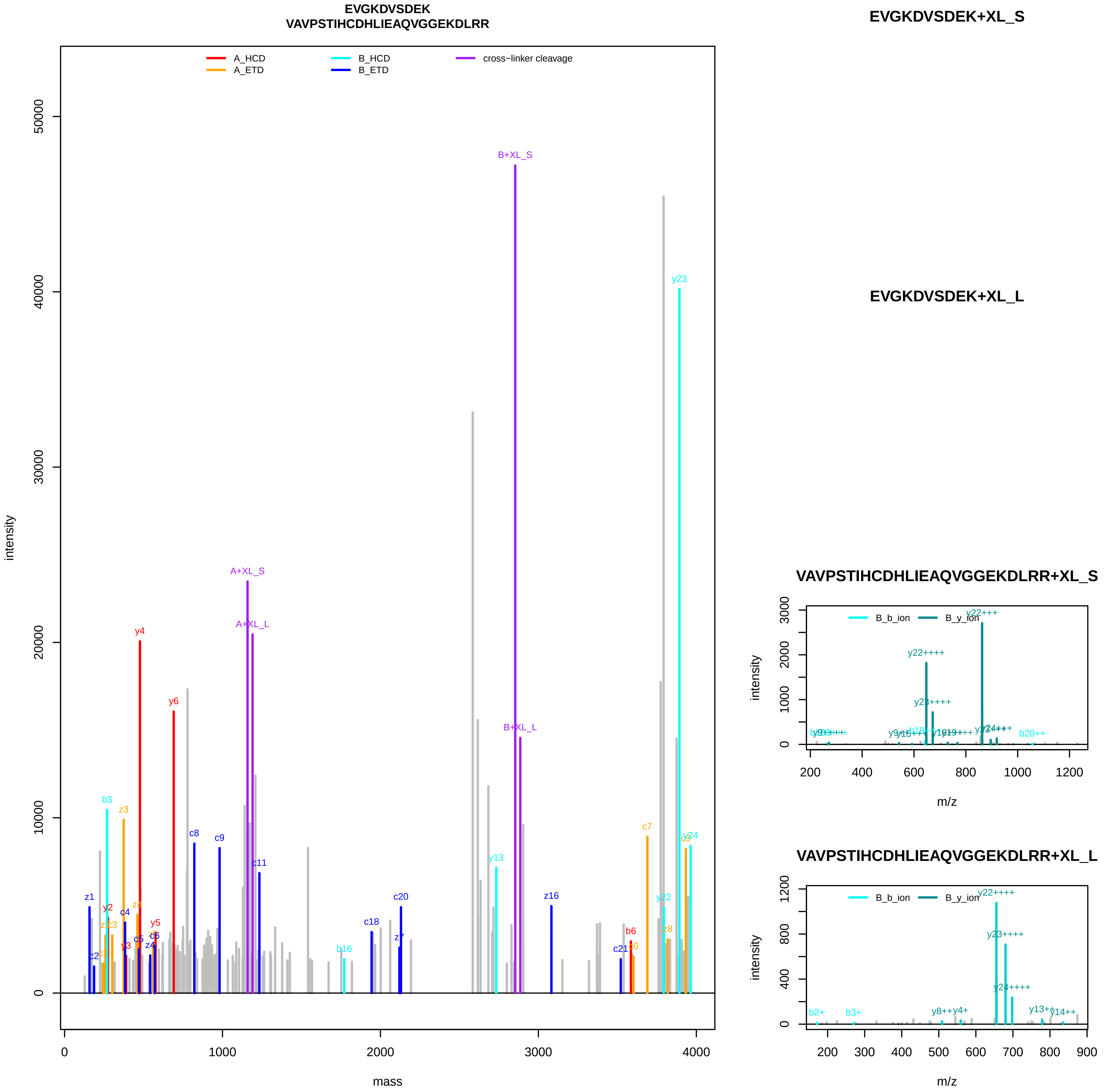
EVGKDVSDEK
VAVPSTIHCDHLIEAQVGGEKDLRR
A_HCD
A_ETD
B_HCD
B_ETD
cross−linker cleavage
intensity
mass
0
10000
20000
30000
40000
50000
0
1000
2000
3000
4000
B+XL_S
B+XL_L
A+XL_S
A+XL_L
y23
y24
y22
y13
y4
y6
y5
y3
y2
b3
b16
b6
z3
z4
z8
c7
c9
c8
c9
c11
c4
c5
c6
c2
c18
c20
c21
z1
z4
z7
z16
EVGKDVSDEK+XL_S
EVGKDVSDEK+XL_L
VAVPSTIHCDHLIEAQVGGEKDLRR+XL_S
B_b_ion
B_y_ion
y22+++
y22++++
y23++++
y24+++
b20++
m/z
VAVPSTIHCDHLIEAQVGGEKDLRR+XL_L
y22++++
y23++++
y24++++
y8++y4+
y13++
y14++
b2+
b3+
m/z
